# Pulmonary Consumption

**Published:** 1878-12

**Authors:** Willis E. Ford

**Affiliations:** Assistant Physician N. Y. State Lunatic Asylum, Utica, N. Y.


BUFFALO

### Medical and Surgical Journal.

VOL. XVIII. DECEMBER, 1878. NO. 5.

### Original Communications.

:o:

*ART. I.—Pulmonary Consumption.* By WILLIS E. FORD, M. D., Assistant Physician N. Y. State Lunatic Asylum, Utica, N. Y. Read before the Oneida County Medical Society, October 8th, 1878.

It is estimated that pulmonary consumption is the cause of one-seventh of all the deaths that occur. The census reports show that from 1850 to 1870, the general death rate diminished considerably, though the proportion of deaths from consumption, as compared with all other causes, was larger in 1870 than in 1850. In 1870, there were 253 deaths from consumption to every 100,000 inhabitants in the New England States. These statistics also show that in the North-Eastern States consumption is more prevalent on the sea board, along the shores of Lake Erie, Lake Ontario and the St. Lawrence, and in the Mohawk Valley. It is less prevalent in Eastern and Southern New York, and west of the Connecticut river In our own city during the last two years, more than fourteen per cent. of all deaths were from consumption. This is truly a terrible exhibit of facts, and this scourge, more to be dreaded than cholera or yellow fever, justly claims a large share of the attention of physicians.

Of late years, real advances have been made in our knowledge of the nature and proper management of the disease—advances due in part to more accurate statistics of a large number of cases from which deductions could be made as to the influence of climate, of certain occupations, character of soil in various localities, etc.; while it is largely due to a more accurate knowledge of the pathology of the disease.

I think I may safely say that the old theory that tubercle is the sole cause of chronic degenerative diseases of the lungs, has lost the support of the larger part of the profession, and that the inflammatory nature of most of these diseases is pretty thoroughly established. To the Germans we are largely indebted for the original investigations that turned us toward this latter theory. The art of physical diagnosis now enables us to detect slight changes in the lungs and to be tolerably certain as to the character of these changes, and therefore enables us to make distinctions, that a few years ago were impossible.

If it be true that most of the cases of consumption have their origin in local and inflammatory processes, and not in the insidious deposit of new formation cells, a result of inheritance; then we must believe that early treatment and proper care will effect a cure in a certain number of cases, and in other cases will arrest the progress of the disease and prevent its full development.

There is a vast amount of literature on this subject, and I think that any one who will take the pains to look it up, will be convinced that the preponderance of evidence tends to establish the inflammatory nature of the disease, and that of late, the number of cases found to be strictly tubercular, and therefore hopeless from the first, has been steadily diminishing.

The processes of inflammation are so varied and result in deposits which may afterward undergo so many changes, that they may explain all the morbid appearances found in the lungs of those who have died of phthisis in any of its stages, excepting in a few rare cases. It seems to me that the general acceptance of this theory on the part of physicians is a matter of great importance, not only as regards the treatment of recognized cases of developing phthisis, but also as regards the prevention of the trouble altogether. Reinhardt asserted as early as in 1847, that the so-called tubercle may originate from pus cells, and thereby he deprived it of importance. By 1850, he had established the fact that many substances hitherto regarded as tuberculous, were identical with inflammatory products. At about this time Virchow began to teach that caseous metamorphosis, the tubercularization, was a general process of necrobiosis of tissues. He limited the term tubercle to the miliary tubercle, which he still regards as a new formation. Baylis' view is that the miliary tubercle plays but a secondary part in phthisis, is developed in the later stages of the disease, and is rapid in its course when once the deposit begins. Niemeyer, whose teachings have done so much toward the advancement of our knowledge of this disease, holds the same view. Carswell, a writer of note, describes the same forms of phthisis. In this country, Dr. A. L. Loomis has for years taught that phthisis, aside from miliary tuberculosis, has a local and inflammatory origin, and in its early stages is, therefore curable, and I think that among the best observers this belief is now pretty generally entertained.

As to the causation of consumption, we can safely say, the more we are assured of its local and inflammatory nature, the less importance does hereditary transmission assume. Niemeyer says: "I do not hesitate to say, in spite of all assertions to the contrary, that it is by no means sufficiently proved that tuberculosis, in the strictest sense, is an inheritable disease." Again: "Quite as decidedly as we have opposed the evidence that tuberculosis is inheritable, must we pronounce in favor of a frequent occurrence of an inherited disposition to pulmonary phthisis." Ruehle, in Ziemssens' Cyclopedia, advocates the theory of the inheritability of phthisis, but says: "I believe that systematic and persistent observation of judicious rules of life may overcome such an inherited tendency." In the report of the State Board of Health of Massachuetts for 1873, a valuable document, is given the opinions of 210 physicians from different parts of the State, on the main points of interest concerning consumption. To the first question, "Is consumption caused or promoted by hereditary influences," 205 answered in the affirmative, one in the negative, and four did not declare. To the second question, "Can consumption apparently be prevented from occurring in children hereditarily predisposed to the malady?" 126 answered in the affirmative, 20 in the negative, 15 that it "can be retarded," 10 were doubtful, and 45 did not declare. These statements from the same men, would seem to be somewhat conflicting so far as the real first cause of the malady is concerned. Common observation shows that the children of parents enfeebled by this slowly exhausting disease, are liable to be weak, to have but little power of resisting the encroachments of disease, and therefore, to fall an easy prey to consumption. This is equally true, however, concerning the offspring of parents suffering from any other progressive and equally exhausting disease, and also regarding the children of drunkards. It is by no means true that the majority of children of phthisical parents die of the same disease. These facts should of course induce consumptive parents so unfortunate as to have children, to give them the best hygienic surroundings, and it is the duty of the physician to assure them that with proper care the disease may be avoided.

The common impression that "catching cold" is the great cause of consumption, is not warranted by the facts. Those influences however, that are followed by bronchial catarrh and hyperæmia of the lungs, and all slow pneumonic processes, are most dangerous. I need only mention mechanical irritation, caused by the inhalation of irritating substances, such as coal dust, copper and iron filings, or the irritation and inflammation caused by retention of blood in the air cells after a bronchial or pulmonary hemorrhage. I should like to say, however, that many hemorrhages ought to be looked upon as causes of phthisis rather than the evidence of unrecognized phthisis.

A chapter might be written and profitably discussed here, on the influence of excessive muscular strain and unusual muscular development in causing consumption. In these times when physical training is carried to such an excess by many, and great muscular development is said to prevent disease, it is certainly worth while to call attention to the fact that men who keep themselves in long and severe training, and reach a condition of great muscular development, are extremely liable to lung disease. The proportion of prize fighters, lifters of heavy weights, leapers, etc., who die of this disease is very large. The severe and oft-repeated congestions of the lungs, caused by muscular strain or prolonged physical exercise, not unfrequently results in a hemorrhage at the time, or the capillaries of the lungs may be so engorged as to never be able to return to their normal condition. The number of persons injured by the health-lift is not small, while its originator, a Harvard man, who reached an enormous muscular development, has since died of consumption.

Here we may mention also the pernicious practice of inflating the lungs to their utmost capacity, and of striking the chest with the fist in order to "strengthen the lungs," indulged in by weak people.

The question of climate is a very important one. It was formerly thought that a warm and dry atmosphere were the essentials. It is now known, however, that cold is not to be dreaded, nor does the amount of rainfall of a region seem to exercise any marked influence in the production of the disease. That kind of moisture which is caused by evaporation is most deleterious, however. Laennec states that the dampness arising from such soil as prevents perfect drainage, is one of the most certain developing causes of phthisis, and he mentions a locality having such a soil in which the dampness was so constant, and of such a character that more than two-thirds of the inhabitants died of phthisis. Dr. Bowditch, of Boston, has made a careful comparison of statistics furnished by physicians in all parts of Massachusetts, and from this, shows that among the prominent causes of consumption is exposure to soil moisture. In England, in 1865 and 1866, inquiries were made under Government authority, into the effects of drainage works, and other sanitary regulations designed to promote public health. Twenty-four towns were selected in which improved drainage had been established, and it appears that while the general death rate was smaller, the proportion of deaths from consumption had been strikingly diminished. The chief medical officer in his report says "the noted and most important conclusion suggests itself, that the drying of the soil, which in most cases accompanied the laying of main sewers in the improved towns, has led to the diminution, more or less considerable, of phthisis." In this connection one cannot help wondering if in this city, with the extraordinarily high percentage of deaths from phthisis, something more could not be done to prevent the scourge.

Statistics show that a soil composed of sand or gravel or porous rocks, gives to the inhabitants of the region comparative immunity from the disease, as compared with a clay soil, or one composed of impervious rock, which allows all the water collected to evaporate back again into the air.

Mere altitude alone seems to exercise but little influence on the advent or course of the disease. While it cannot be denied that regions at an elevation of 1,800 feet above the sea level are generally more free from the disease, yet it is not so much due to a lessened atmospheric pressure as it is to the purity of the air, its freedom from dust and irritating substances. Dr. Loomis says: "Experience has shown me no place where phthisical invalids in all stages of the disease do worse than among the Catskill Mountains. Without exception, in those phthisical invalids under my observation who have resorted to this mountain range, the disease has made much more progress than in any other locality. I find similar testimony given by other observers in regard to other mountain regions."

In general, all causes that tend to depress vitality may act as predisposing causes of consumption. Poor and insufficient food, overwork, confinement in over-crowded, dusty and ill-ventilated rooms, and especially all conditions tending to exhaustion of the nervous system, favor the advent of this disease. Every one familiar with hospital practice must have observed the unusually large number of deaths from phthisis among patients with chronic nervous disorders, and also among those given to certain excesses tending to rapid exhaustion of nervous force. I was surprised when in a general hospital to observe that the common cause of death among the large number of prostitutes treated there, was consumption.

We may group all cases of phthisis under three general heads: acute miliary tuberculosis, catarrhal phthisis, and fibrous phthisis.

Acute miliary tuberculosis is a comparatively rare disease, and runs its course in about six weeks. The symptoms are rather obscure and have a strong resemblance to typhoid fever. The development of miliary tubercles in the course of an ordinary phthisis, is not rare, and Niemeyer says: "The possibility, that in the course of a pulmonary phthisis caused by pneumonic processes a tuberculosis may be developed, must be constantly kept in view." And again: "The greatest danger to most phthisical patients is the development of tubercles." I am very fortunate to-day in being able to present you some fine specimens of miliary tubercles, and desire to call your attention to the specimens mounted by Dr. Deecke for microscopic observation. A careful comparison of the specimens before you will, I think, convince every one of the radical difference between disease due to this neoplasm and the common forms of consumption.

The following case of acute miliary tuberculosis is rather a typical one. A man aged 23, a laborer, came into my ward in Hospital in the summer of 1873 with a history of two weeks illness. His condition then, together with his history, pointed to typhoid fever, or at least to some continued fever. He had a temperature of 103 and a pulse of 120, and though he was a strongly built man and in good flesh at the time, he was greatly prostrated, and his face showed great anxiety. His breathing was labored and about 24 to the minute, but an examination of his lungs failed to reveal anything very definite, more than a slight roughness of the smaller bronchi and a wavy inspiration. His temperature for a week did not vary much, and his symptoms did not change, and we were able to exclude the fevers. After this he coughed a little, and in the third week his breathing was more labored, and his temperature ranged higher. He lived something over a month after coming into the Hospital, and his entire illness only lasted from six weeks to two months. I am unable to give the facts regarding heredity in this case. An autopsy revealed miliary tubercles, and in the lung though all the tissue was closely studded with them, there was no large aggregations which could indicate the real nature of the disease during life. There was no cavity in the lung, and the appearance throughout was about like the specimen of miliary tubercle before you.

This specimen of miliary tubercle is from a patient with a similar history. A man aged 29, of good physical development, without hereditary taint, with no history of previous lung trouble, began to feel badly and took to the bed about the first of February last. He had no chill, no cough, and when his temperature began to rise his physician was somewhat puzzled to explain his condition. He died on the third of March, and it was only a short time before his death that his real condition was suspected. There were no large aggregregations of tubercles, there were no cavities in the lungs, nor were there any other unusual appearances, excepting miliary tubercles filling the lungs and scattered through other organs. You will observe under the microscope that certain of the tubercles are nearly spherical, and show concentric layers composed of a material somewhat resembling pavement epithelium. These seem to be the younger formations while other spots are irregular in outline, and seem to be accretions of several small growths, and you will notice that some of these are breaking down from the centre. The mounted specimens before you—taken from other organs have precisely the same outline, the same size and the same structure. These neoplasms differ in structure from that of any normal tissue and from all other pathological conditions. They are so distinctive that it is impossible that the masses found in the lungs of phthisical patients ordinarily could be mistaken for these new formation cells.

In catarrhal phthisis the primary changes occur in the cavities of the air cells and the bronchi, and are epithelial and cellular in their nature.

In fibrous phthisis the primary changes occur in the bronchial and alveolar connective tissues and are connective tissue hyperplasias. These are the ordinary forms of consumption, and are undoubtedly local in their origin. The specimens before you will illustrate almost every stage of the disease.

In June last, I had the opportunity of making an autopsy on a phthisical patient, aged 60, and I am able to-day to present you the specimens together with a very accurate history of her illness.

As early as 1852 the patient had a pulmonary or bronchial hemorrhage. She was strong and vigorous, and of a healthy family, and under good treatment seemed to recover. There remained some bronchial catarrh however, and she always had a winter cough. She got along very well, and was able to attend to her household duties up to 1869, when she had another hemorrhage, and consolidation of lower lobe of lung followed. Then began a slow process of breaking down of lung tissues. Fine rales could be heard about the spot of consolidation, and expectoration was profuse; stimulation, good diet, and quinine was the treatment which was persisted in for more than a year, the disease seemed to limit itself and her symptoms, excepting dyspnœa gradually improved. Hypertrophy of the heart followed. She was never very strong after this, though able to be about the house most of the time. She had a cough and great dyspnœa on the slightest exertion during the remainder of her life, though she continued in fair flesh. Her death was sudden.

An autopsy revealed an extensive sack of yellowish creamy matter occupying nearly the whole of the left thoracic cavity. This fluid measured 20 ounces, was not offensive, and as we opened the sack a drachm or more of clear oil floated on the top. I give below an analysis of the fluid, made by Dr. Deecke:

Water 16 ounces, and solid matter 3¼ ounces.

The solid matter consisted of:

| | | |
|---|---|---|
| Phosphate of Lime . . . . . | 1¼ | ounces, |
| Chloride of sodium, . . . . | 41 | grains, |
| Grape Sugar, . . . . . . | 21 | " |
| Cholesterine, . . . . . | 1⅛ | ounces, |
| Caseine, . . . . . . . | 34 | grains, |
| Fat, . . . . . . . . | 126 | " |
| Dry Albumen, . . . . . . | 16 | " |
| Insoluble inorganic matter, . . . . | 44 | " |

A portion of the thick, well formed membrane which enclosed this mass is here exhibited.

I think this case especially interesting as showing the conservative tendency of the disease. The patient had repeated hemorrhages, and finally extensive destruction of lung tissue, and yet the process limited itself—the products of inflammation became encysted and innocuous, and her life was prolonged for many years.

Another case now under observation is that of a woman aged 51, who, on the 15th of March, 1875, had a chill, and the usual symptoms of pneumonia. At 2 P. M. on the 17th, her temperature reached its maximum, 104½ degrees. The lower and middle lobes of the right lung were affected. After running an ordinary course, resolution was not completed in the middle lobe, and there remained a chronic catarrhal pneumonia. Some six months after the first attack, a cavity was formed, after which the extent of bronchial catarrh was somewhat less. Cicatrization has taken place, and the patient is now in a very comfortable condition, though there is a slight cough and a considerable dyspnœa on exertion. She has gone through one attack of insanity since then, and recovered without any extension of lung trouble. The treatment in this case during the latter period of resolution from pneumonia, and during the slow degenerative changes later, was quinine, and stimulation carried to the extent of reducing the temperature and preventing night sweating.

I will here mention a case which was under my care for some time, similar to the last in the advent and course of the pneumonia, excepting that resolution did not begin in the middle lobe (the right lung only as in the previous case was affected) and dullness and absence of respiratory murmur continued for more than two months. The temperature during this time ranged from 102 to 104 degrees, and we were assured there was the formation of a large amount of pus. He took during some of this time a quart of brandy a day. Suddenly one day, during a paroxysm of coughing, he expelled a plug from the large bronchus leading to the affected lobe, and immediately there gushed out more than a pint of offensive pus. The plug I saw, and it was cylindrical, about two inches in length, by three-quarters of an inch in diameter, composed of exfoliated mucous membrane and inspissated mucous.

His temperature immediately went down, his appetite returned, and convalescence began. There remained some bronchial catarrh for a few months, but this soon subsided.

This case is a very interesting one, and unique so far as I know, and I believe his recovery was largely due to the use of alcohol, in preventing rapid tissue changes, and especially to its local action in hardening the tissues as it was exhaled in the breath; thus avoiding extensive degeneration of lung tissue.

Another case of recovery was that of a man aged 33, an apothecary, without hereditary taint, who some ten years ago had pleurisy of the right side, and recovered and remained well up to

December, 1875. He was then somewhat run down in general health, was confined rather closely to his business and worked hard. He had had some pain under his right clavical for a few days, and a dry irritating cough, and one morning before getting up, began to spit blood. The hemorrhage was not a large one, but the patient was much prostrated afterwards, and when I saw him a few days later he had a temperature of 102, with a pulse of 110, and was restless, sleepless, and much depressed about his condition. The site of the trouble was in the apex of the right lung, and a catarrhal pneumonic process followed with profuse muco-purulent sputa, night sweats, and a temperature ranging from 100 to 102 degrees. A very appreciable consolidation of lung tissue resulted, but no further hemorrhage occurred and after two months the patient's general condition began to improve. He was so thoroughly impressed with the necessity of proper nutrition and care that he did not require urging to continue his treatment for nearly a year. The consolidation disappeared by a slow process of liquifaction and absorption from the circumference, and for a year and a half he has been healthy and strong.

There is no doubt but that many of these cases have a tendency to self limitation and to repair. The judicious use of quinine in cases where there is a rapid formation of pus, or rapid cell proliferation, promises much in retarding the processes of the inflammation and preventing serious damage to lung tissue. It seems to me that the discriminate, and yet in some cases, very free administration of alcohol promises more than we have been accustomed to think it capable of accomplishing. In lowering temperature, in retarding retrograde metamorphosis, thus taking temporarily the place of food, it has long been recognized as an efficient agent. But it is reasonable to suppose that in some cases the local action of alcohol in hardening the tissues through which it passes, from the thin walls of the capillaries of the lungs into the breath, may prevent the softening of the tissue and prevent the formation of extensive cavities. Alcohol undergoes no digestive changes within the body, and is therefore as active when exhaled from the lungs as if it had never been within the body. In cases of rapid emaciation in connection with a high temperature, the inunction of the whole body with olive oil or cod liver oil, gives a vast amount of comfort and prevents, in a measure, the rapid wasting. This practice of inunction so common among the ancients, is less popular now than it ought to be.

The practice of sending phthisical patients away in search of health is becoming so common, that the question of locality is a very important one. The ancient custom was for phthisical patients to go to the sea shore. We now know that this is the worst place in the world for those in whom we wish to arrest the process of breaking down of tissue. Only those advanced in life or where the slow process taking place within the lung needs stimulating, do well at the sea shore. Of late the fashion has been to resort to the mountains, though the indications in every case are by no means met by the change. Certain catarrhal conditions are benefitted by mountain air, though it is probable that the purity of the air in these regions, its freedom from germs of all kinds, is the one all important restorative agent and that mere altitude is of secondary importance. In selecting a locality for those inclined to develop this disease, the important consideration should undoubtedly be that of soil. Freedom from soil moisture is a feature of any locality that should especially recommend it. It has long been known that phthiscal patients do well in the midst of pine forests. It was thought that the inhalation of air in which the balsams of these evergreen trees could be detected, exerted a curative influence. More recent investigations have shown however, that the turpentine exhaled from these pine or hemlock forests possesses the power to a greater degree than any other known substance of setting ozone free. Ozone itself, inhaled into the lungs possesses no curative power, but it does destroy the germs of the atmosphere, and noxious gasses, and thus renders the air especially salubrious. On this account the pine regions of Georgia and North Carolina have become of late popular resorts for those with affections of the lungs.

There must be a careful discrimination of cases, however, for the profession is by no means agreed upon the question of climate, and many invalids are sent away every year who would be far better off at home. Nothing can be expected from a change of climate with patients having true tuberculosis, and it is cruel to deprive persons in the later stages of phthisis of the comforts of their homes. In the fourth biennial report of the Board of Health of California, for the years 1876 and 1877, Dr. Hatch, the permanent secretary of the board, has a valuable paper on the "Relations of the Climate of California to Consumption." He says: "The premonitory stage of phthisis, or the first stage of its actual development, is the only one in which climate may be safely relied upon. That some cases in the second stage may be greatly benefited, especially when the nutritive processes are not seriously impaired, that a few may secure an apparently permanent arrest of the disease, and enjoy good health for many years; but that the climate of California, while it may for a time seem to inspire hope, offers in reality no very strong inducement to those lapsing, or who have already passed into the third stage of the disease. That the remedy, if found beneficial, must be continued from year to year until the restoration of the nutritive processes is complete, and the progress of disease, as determined by physical signs, appears to be arrested."

